# Dataset of manually classified images obtained from a construction site

**DOI:** 10.1016/j.dib.2022.108042

**Published:** 2022-03-12

**Authors:** Alexandre Del Savio, Ana Luna, Daniel Cárdenas-Salas, Mónica Vergara, Gianella Urday

**Affiliations:** Faculty of Engineering and Architecture, Universidad de Lima, Peru

**Keywords:** Construction images, neural network training images, construction monitoring images, construction objects, computer vision image classification

## Abstract

A manually classified dataset of images obtained by four static cameras located around a construction site is presented. Eight object classes, typically found in a construction environment, were considered. The dataset consists of 1046 images selected from video footage by a frame extraction algorithm and txt files containing the objects' class and coordinates information. These data can be used to develop computer vision techniques in the engineering and construction fields.

## Specifications Table

**Table:** 

Subject	Engineering
Specific subject area	Civil and Structural Engineering, Image Classification, Computer Vision
Type of data	Images.jpg Archives .txt
How data were acquired	The data were acquired by four static cameras located around a construction site: one bullet type camera, model IPC-HFW2831T-ZS-S2, and three motorized IP PTZ cameras, model SD10A848WA-HNF. The cameras were programmed using the DSSExpress-Base-License software [4], and for image classification the LabelImg v.1.8.1 tool [3] was used.
Data format	Raw and filtered (jpg, txt).
Parameters for data collection	Four static cameras were located at distances ranging from 5 to 90 meters, with heights varying from 15 to 55 meters. The images were taken from November 2020 to January 2021, between 08:00 and 12:00 hours.
Description of data collection	The data was collected by executing a frame extraction algorithm applied to the videos provided by the surveillance cameras.
Data source location	All images were acquired from the construction project of the University Wellness Center located at the Universidad de Lima, Lima, Peru. Lat. –12.084307°, Long. –76.971031°
Data accessibility	The data is hosted on a public and trusted repository. Repository name: Repositorio Institucional – Universidad de Lima. Direct URL to data: https://doi.org/10.26439/ulima.datasets.13359 The algorithm corresponding to the extraction of frames has been provided publicly through the GitHub repository [2].

## Value of the Data

• This data provides 1046 manually classified images and their corresponding .txt classification files, including 8 types of objects (Table 1) found in a construction site. The images were taken from four cameras placed at different points, at different moments, around a construction site.

**Table 1:** Classes used for manual classification. Adapted from [1].

ID	Classes	Objects
0	Dump_truck	
1	Excavator	
2	Concrete_mixer_truck	
3	Skid_steer	
4	Tower_crane	
5	Truck_crane	
6	Truck	
7	Person
• The data presented is useful for researchers who wish to use or add these classified images to their databases, for further classification and object detection training through construction monitoring systems with computer vision.
• This dataset can be used to validate a neural network of object recognition.
• This dataset contains images that are specific to construction sites, focusing on both machinery and construction personnel.

## 1 Data Description

**Fig. 1:**
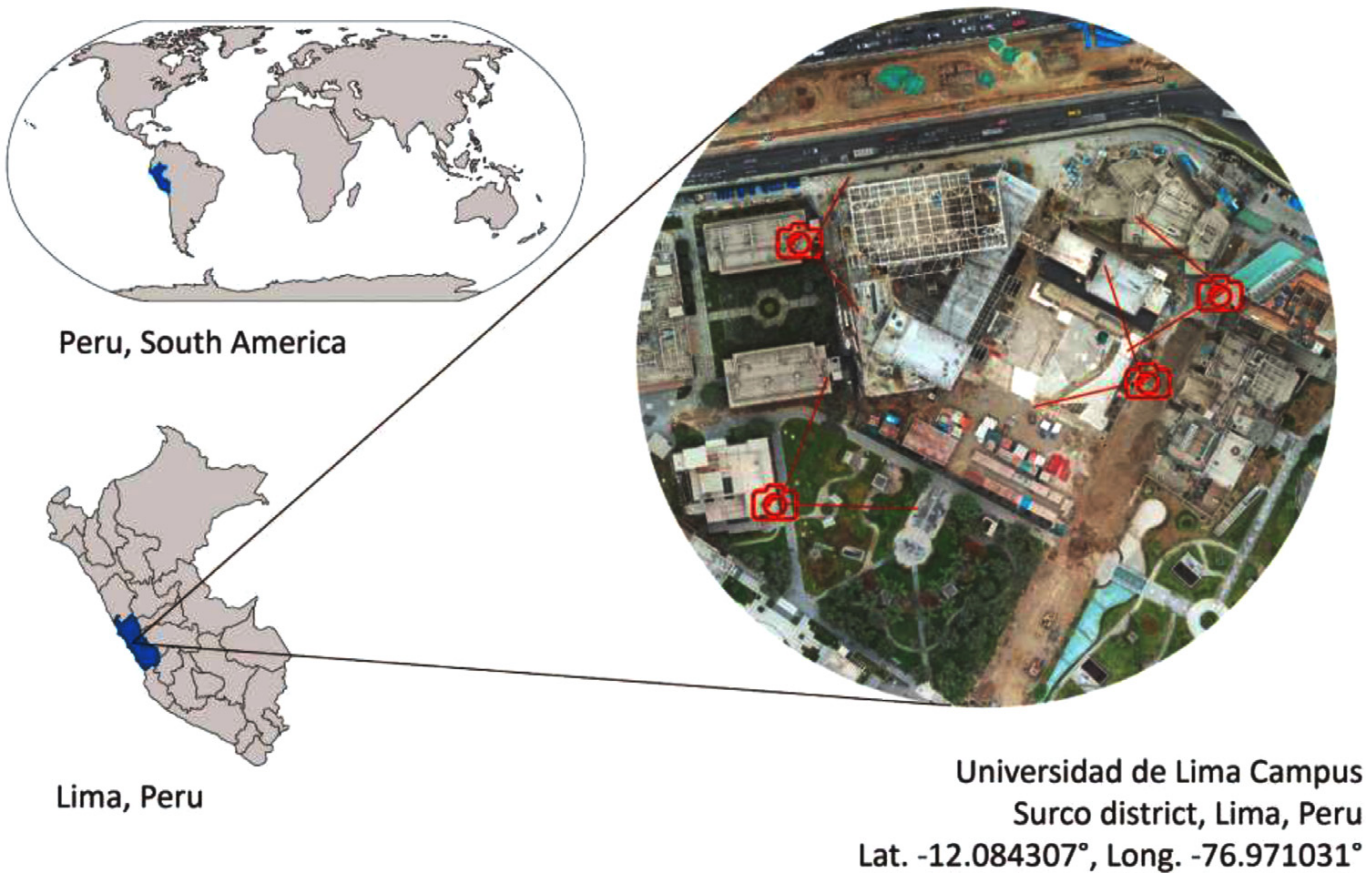
Construction site location. Adapted from [1].

Images of the construction site for the University Wellness Center located at Universidad de Lima were obtained by four static cameras video footage (Fig. 1). A total of 1046 images were collected. This data was verified by Del Savio et al. [1] to use artificial intelligence in object detection in a construction site.

Table 1 shows the classes used for manual classification and their respective images. These elements are found in the classes.txt file. As the elements are part of a Python list, the ID starts with the number 0 for element 1, and finishes with ID 7 for element 8.

**Fig. 2:**
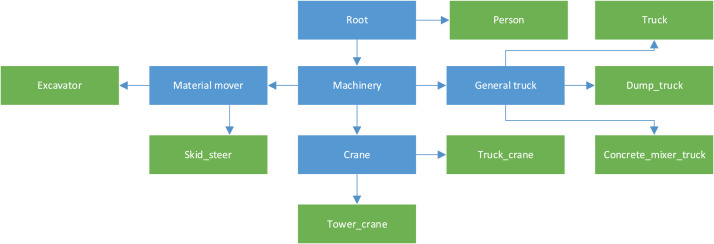
Hierarchical classification proposed.

These classes could be further classified in groups according to the needs of the research, particularly in cases where our granularity level is not desired, as per the example shown in Fig. 2. This hierarchical classification proposed, however, was not applied to the dataset.

Table 2 shows two examples used for the manual classification: the original images, the images during the manual classification, and the results, in .txt format, after the process. The first column presents the images obtained through the execution of the frame extraction algorithm, from the videos of the surveillance tools. The images are in jpg format, with 3840 × 2160 pixels. The algorithm corresponding to the extraction of frames has been provided publicly through the GitHub repository [2]. The second column shows the pictures of the construction site during the manual classification process, with the LabelImg v.1.8.1 software [3]. The objects to be classified were indicated in the green quadrilaterals around them. Finally, the results of their respective manual classification are shown in the third column. The archives are in.txt format and the structure is: the first column is the ID of the class classified (Table 1), the second and third columns are the X and Y coordinates of the beginning of the selection, and the fourth and fifth columns are the X and Y coordinates of the end of the selection of the green quadrilateral.

**Table 2:** Examples of selected images before, during and after classification.

Original image	Images during classification	Results in .txt format
		
		

**Fig. 3:**
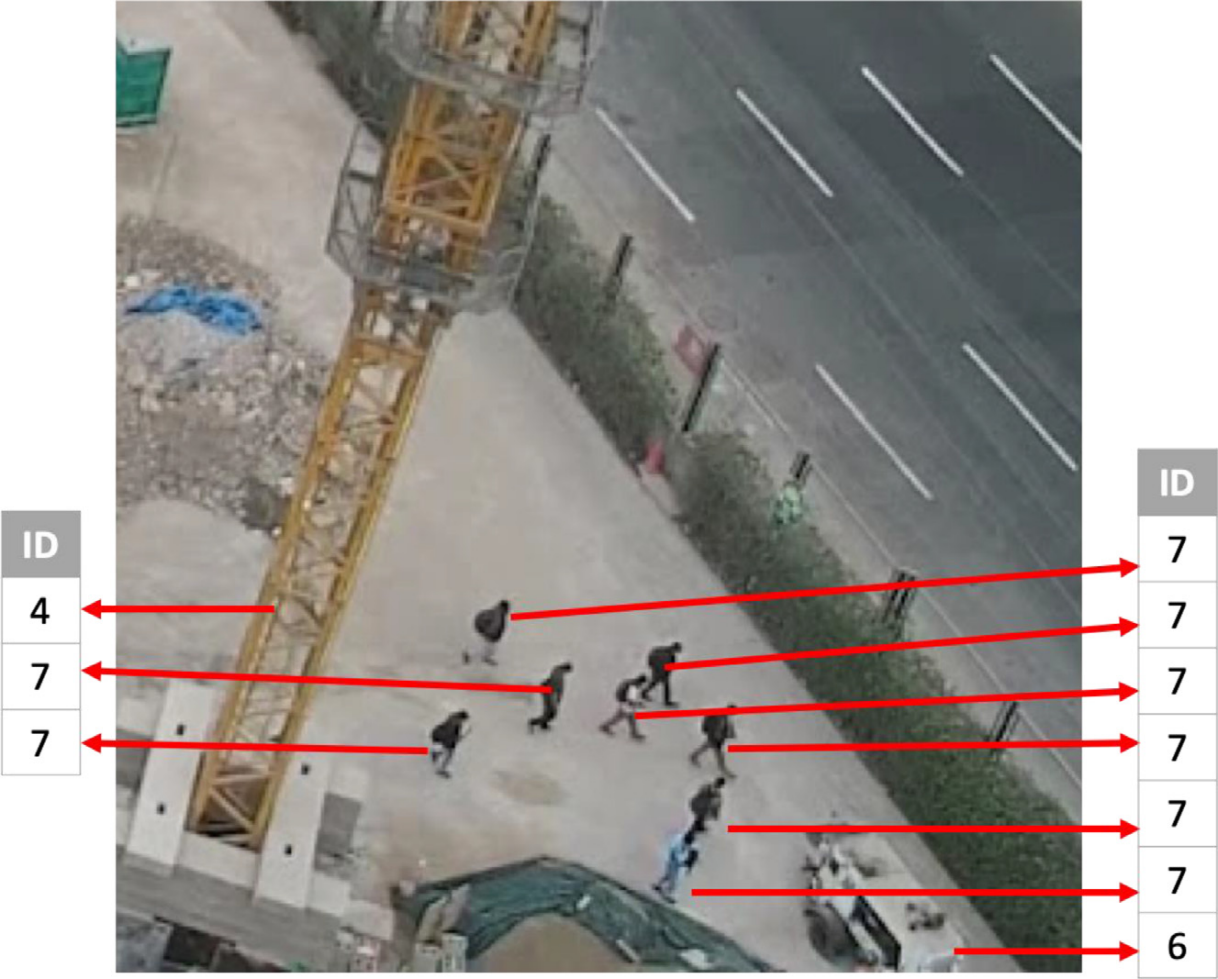
Extract of IMG100.jpg's objects with their respective IDs.

Fig. 3 shows a section from IMG100.jpg (Table 2) where the objects are linked to their respective ID, according to Table 1.

## 2 Experimental Design, Materials and Methods

**Fig. 4:**
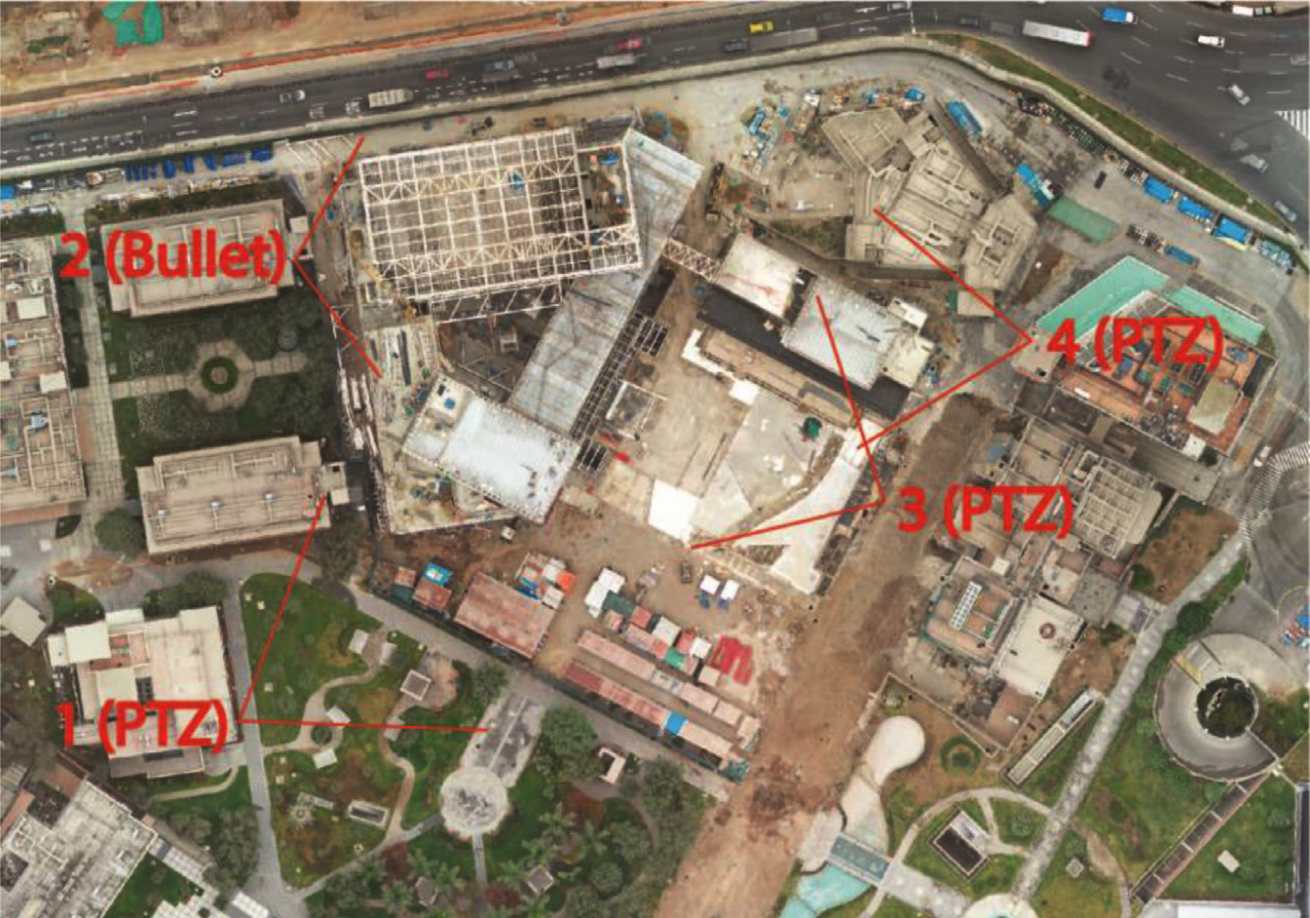
Location of static cameras in construction site.

This dataset was gathered using four static cameras: a bullet-type camera (Dahua Technology, 8MP Lite IR Vari-focal Bullet Network Camera) and three motorized IP PTZ cameras (Dahua Technology, 4K 48x Starlight+ IR WizMind Network PTZ Camera). These cameras recorded video footage from four different points of view around the construction site (Fig. 4) using the video management system DSS Express [4]. The images used were collected between November 2020 and February 2021, Table 3 shows the date and time, according to the ID of the image Table 4. shows an average of the weather conditions during the dates of the collected images, describing the illuminance and air temperature.

**Table 3:** Date and time of images.

Image ID	Date (MM, DD, YY)	Time (24 hrs)
IMG1	December 02, 2020	09:10
IMG2 – IMG54	January 06, 2021	10:59 – 11:05
IMG55 – IMG106	December 02, 2020	09:09 – 09:15
IMG107 – IMG157	January 06, 2021	10:59 – 11:05
IMG158 – IMG207	February 12, 2021	08:59 – 09:05
IMG208 – IMG258	November 20, 2020	01:09 – 01-15
IMG259 – IMG260	November 20, 2020	09:05 – 09:05
IMG261 – IMG302	November 20, 2020	11:08 – 11:13
IMG303 – IMG322	November 20, 2020	13:12 – 13:14
IMG33 – IMG361	December 02, 2020	09:11 – 09:15
IMG362 – IMG450	January 06, 2021	11:00 – 11:05
IMG451 – IMG457	November 20, 2020	09:05 – 09:05
IMG458 – IMG562	January 22, 2021	08:36 – 11:10
IMG563 – IMG627	November 20, 2020	08:59 – 09:05
IMG628 – IMG699	November 20, 2020	11:13 – 11:13
IMG700 – IMG764	December 02, 2020	09:09 – 09:15
IMG765 – IMG827	January 06, 2021	10:59 – 11:05
IMG828 – IMG901	February 12, 2021	08:59 – 09:04
IMG902 – IMG969	November 20, 2020	08:59 – 09:05
IMG970 – IMG1043	December 02, 2020	09:10 – 09:10
IMG1044 – IMG1049	January 22, 2021	11:10 – 11:11

**Table 4:** Weather conditions during collected images.

Date (MM, DD, YY)	Time (24 hrs)	Illuminance (lx)	Air temperature (°C)
November 20, 2020	09:05 – 13:15	62000	24
December 02, 2020	09:09 – 09:15	70000	23
January 06, 2021	10:59 – 11:11	32400	22
January 22, 2021	08:36 – 11:10	17500	22
February 12, 2021	08:59 – 09:05	50000	25

A frame extraction algorithm was used to obtain the images from the video footage in jpg in a 3840 × 2160 pixels format, every 200 frames. The images went through a manual classification process with the LabelImg v.1.8.1 software [3] to identify the construction objects on site by creating quadrilaterals around the objects and assigning them a class. The results were exported in a .txt file for each image.

## Ethics Statement

This research did not involve any human subjects, animal experimentation nor social media platforms.

## CRediT authorship contribution statement

**Alexandre Del Savio:** Conceptualization, Validation, Resources, Writing – review & editing, Supervision, Project administration, Funding acquisition. **Ana Luna:** Validation, Formal analysis, Visualization, Resources. **Daniel Cárdenas-Salas:** Methodology, Software, Validation, Formal analysis, Investigation. **Mónica Vergara:** Investigation, Data curation, Writing – original draft, Visualization. **Gianella Urday:** Software, Investigation, Data curation, Writing – original draft.

## Declaration of Competing Interest

The authors declare that they have no known competing financial interests or personal relationships which have or could be perceived to have influenced the work reported in this article.
